# Food as circadian time cue for appetitive behavior

**DOI:** 10.12688/f1000research.20829.1

**Published:** 2020-01-29

**Authors:** Ralph E. Mistlberger

**Affiliations:** 1 Department of Psychology, Simon Fraser University, 8888 University Drive, Burnaby, BC, V5A2S6, Canada

**Keywords:** circadian, food, entrainment, anticipatory activity, ghrelin, ketone, insulin, dopamine, reward

## Abstract

Feeding schedules entrain circadian clocks in multiple brain regions and most peripheral organs and tissues, thereby synchronizing daily rhythms of foraging behavior and physiology with times of day when food is most likely to be found. Entrainment of peripheral clocks to mealtime is accomplished by multiple feeding-related signals, including absorbed nutrients and metabolic hormones, acting in parallel or in series in a tissue-specific fashion. Less is known about the signals that synchronize circadian clocks in the brain with feeding time, some of which are presumed to generate the circadian rhythms of food-anticipatory activity that emerge when food is restricted to a fixed daily mealtime. In this commentary, I consider the possibility that food-anticipatory activity rhythms are driven or entrained by circulating ghrelin, ketone bodies or insulin. While evidence supports the potential of these signals to participate in the induction or amount of food-anticipatory behavior, it falls short of establishing either a necessary or sufficient role or accounting for circadian properties of anticipatory rhythms. The availability of multiple, circulating signals by which circadian oscillators in many brain regions might entrain to mealtime has supported a view that food-anticipatory rhythms of behavior are mediated by a broadly distributed system of clocks. The evidence, however, does not rule out the possibility that multiple peripheral and central food-entrained oscillators and feeding-related signals converge on circadian oscillators in a defined location which ultimately set the phase and gate the expression of anticipatory activity rhythms. A candidate location is the dorsal striatum, a core component of the neural system which mediates reward, motivation and action and which contains circadian oscillators entrainable by food and dopaminergic drugs. Systemic metabolic signals, such as ghrelin, ketones and insulin, may participate in circadian food anticipation to the extent that they modulate dopamine afferents to circadian clocks in this area.

## Overview

In this article, I describe how regularly scheduled meals induce daily rhythms of food-anticipatory behavior by entrainment of circadian clocks. I then consider evidence that these clocks may be located in peripheral organs, such as the stomach, liver or pancreas, or may be entrained by metabolic hormones (ghrelin and insulin) or fuels (ketone bodies) released by these tissues under control of circadian clock genes or in direct response to daily cycles of feeding and fasting. A primary objective is to evaluate how well the evidence accounts for established circadian properties of food-anticipatory rhythms. In reviewing relevant studies, I focus on those experiments that have behavioral endpoints, as these must stand on their own merits, independent of companion experiments that have cellular or molecular endpoints. I conclude with a reminder that animals can anticipate daily rewards other than food, including water, salt, sex and psychomotor stimulants. Evidence for food- and dopamine (DA)-entrainable circadian clocks in the brain’s reward system suggests a common mechanism for anticipation of any salient reward stimulus that recurs at predictable times of day.

## Daily rhythms as circadian clock output

Circadian (~24-hour) rhythms and the clocks that time them are a biosignature of life on earth, an adaptation that enables most organisms to anticipate the regular alternation between day and night and to segregate cellular, systems and behavioral functions in accordance with a diurnal or nocturnal temporal niche. The mechanism of the circadian clock is intracellular and employs interlocking transcription-translation feedback loops (TTFLs) through which the expression of clock genes is activated or repressed by clock proteins ^1^. In mammals, the clock genes *Bmal1* and *Clock* encode proteins that form dimers that bind to enhancer boxes and drive expression of other clock genes, including *Period* ( *Per1*, *Per2* and *Per3*), *Cryptochrome* ( *Cry1* and *Cry2*), *Reverbα* and *Ror* genes. PER and CRY proteins accumulate in the cytoplasm, form dimers, translocate, interfere with BMAL1-CLOCK binding and thereby terminate their own expression. *Bmal1* expression is activated by RORs and repressed by REV-ERBs. Post-translational modifications target PER and CRY proteins for removal, permitting BMAL1-CLOCK to initiate the next cycle of transcription. Post-translational modifications also regulate the period of the cycle ^2^. Clock proteins act as transcription factors for other clock-controlled output genes and thereby propagate circadian cycles through the genome and the functions of the cell ^3, 4^. Cellular inputs that acutely alter clock gene expression or proteins can shift clock phase and enable entrainment to periodic environmental stimuli (Zeitgebers), such as the light–dark (LD) cycle ^5^. The specific clock genes differ across life kingdoms, but the autoregulatory TTFL is a common mechanism. The discovery of clock genes and the TTFL in the fruit fly was celebrated by a Nobel Prize in 2017 ^6, 7^.

In mammals, clock genes exhibit robust circadian cycling in the suprachiasmatic nucleus (SCN), a retinorecipient cell cluster in the basomedial hypothalamus that is essential for entrainment to LD cycles and for circadian organization (that is, “free-running” rhythmicity) in constant environmental conditions ^5, 8^. Clock genes also exhibit circadian expression in other brain regions and in most organs and cell tissues outside of the brain. Bioluminescent gene reporter assays show that clock gene cycling in the SCN and in most non-neural cells and tissues can persist for many cycles, if not indefinitely, *in vitro* ^9– 14^. This has also been established for some other brain regions. Mammalian circadian rhythms therefore reflect the operation of a distributed, multioscillatory circadian system, in which a master clock in the SCN is entrained by LD cycles and drives daily rhythms either directly or by coordinating circadian clocks that control local functions in other brain regions and organs.

## Feeding rhythm as circadian clock input

SCN outputs have a relatively restricted, predominantly hypothalamic, projection field ^15^. Cell groups in these target areas control autonomic and endocrine functions and link the SCN pacemaker to physiology and behavior ^16^. Circadian cycling is further propagated and reinforced by the consequences of behavior. The daily rhythm of feeding and fasting appears to be of special significance ^10, 17^. Circadian clocks in most peripheral organs can be shifted by one or more stimuli associated with feeding rhythms or food intake, including glucose ^18^, cell metabolic sensors (for example, AMP-activated protein kinase ^19^, SIRT1 ^20, 21^ and PARP-1 ^22^) and metabolic and gut hormones (for example, corticosterone ^23^, insulin ^24– 27^ and oxyntomodulin ^28^). Some peripheral clock inputs may be universal, and others tissue-specific.

The distributed design of this system is clearly adaptive; retinal inputs entrain the SCN pacemaker to local time, the SCN determines when animals will eat (assuming food availability is unrestricted), and food intake controls peripheral clocks, ensuring that the organism is physiologically prepared to ingest, digest, absorb, distribute and store nutrients during its waking hours. An interesting feature of this design is that if food availability is restricted to a particular time of day or night, peripheral clocks shift their phase to preserve alignment with mealtime, whereas the SCN pacemaker remains synchronized to LD ^29– 31^. The circadian physiological program is therefore flexible to the extent that it can accommodate even a reversed feeding cycle without forcing the SCN pacemaker out of phase with the LD cycle, thereby potentially preserving SCN-based encoding of daylength ^32^.

For circadian reprogramming of physiology to be useful, mealtime must also control behavior, so that animals are awake and motivated to seek and ingest food when it is most likely to be found. If the SCN pacemaker controls the sleep–wake cycle but does not appreciably change its phase in LD despite inversion of the feeding cycle, then it should oppose foraging activity. There are two possible solutions: either SCN outputs representing circadian phase can be used as discriminative cues that acquire incentive properties when regularly paired with food (an interoceptive “bell” for Pavlov’s hungry dog) or there is another timing device that regulates foraging activity and supersedes SCN output when the two devices send conflicting messages.

## Separate clocks for food- and light-entrained behavior

Long before the word “circadian” was coined and the concept of the circadian clock was firmly established, the biopsychologist Curt P. Richter showed that rats maintained in constant light for many days and fed for only 25 minutes at a fixed time each day exhibited a daily rhythm of locomotor activity that rose to a peak at the scheduled mealtime ^33^. He called this food anticipation. The activity rhythm persisted (albeit weakly) for a few days of total food deprivation, suggesting that anticipation was timed in a “clock-like” fashion and was not merely a reflection of accumulating hunger since the last feeding. Decades later, biopsychologist Robert C. Bolles and colleagues demonstrated that the anticipation rhythm also emerges in rats entrained to LD and fed in the middle of either the day or the night. The authors confirmed that the rhythm persists during total food deprivation ^34– 36^, and most importantly, showed that the anticipation rhythm does not emerge if the feeding schedule is very different from 24 hours (for example, 19 or 29 hours) ^35, 36^. The mechanism was inherently “bound up with the 24-hour cycle”. Two groups ^37– 39^ then showed that the mechanism did not require the SCN pacemaker; rats made arrhythmic by SCN ablation, and maintained in constant dark or light, exhibited robust daily rhythms of food-anticipatory activity (FAA) if fed once daily. Analysis of the formal properties of the rhythm were consistent with control by a circadian clock entrained by food, a so-called “food-entrainable oscillator” (FEO); the rhythm had circadian “limits to entrainment”, shifted gradually rather than immediately following meal shifts, and persisted for as many 24-hour cycles of food deprivation as could be safely tested ^40– 44^. Bolles and Moot had also shown that rats could anticipate two daily meals separated by 6 hours ^34^; Stephan confirmed this in SCN-ablated rats and provided suggestive evidence for concurrent anticipation of two daily meals: one recurring every 24 hours and the other every 25 hours ^44, 45^. This is not unlike activity rhythms driven by the SCN pacemaker, which can also split into two oscillating components that, under some conditions, can free-run separately and may normally couple to sunset and sunrise (communicating daylength by their phase angle difference) ^46, 47^.

These results have been widely interpreted as evidence that mammalian behavior is regulated by two circadian clocks—one (the SCN) specialized for entrainment to LD cycles and the other (somewhere else) for entrainment to daily feeding cycles—and that both clocks are composed of multiple clock cells that can be configured into at least two stable, entrainable groups. Given that the SCN is in the hypothalamus and receives light information directly from the retina, a reasonable (by analogy) working hypothesis is that FEOs for behavior would also be hypothalamic, but located within those cell groups that respond to peripheral metabolic signals and regulate feeding and metabolism. Some hypothalamic lesions and gene knockouts (KOs), targeting defined brain regions, cell types, or receptors in the hypothalamus, are associated with an altered food anticipation phenotype, most often characterized by a lower-level or slower emergence of anticipatory activity or, in some cases, by enhanced anticipation ^48– 51^. Similar phenotypes may follow lesions or KOs targeting brainstem and forebrain areas ^52– 54^.

Interpreting these phenotypes is challenging. The site of action could be upstream or downstream from the clock mechanism, as many factors (sensory, motor and motivational) determine the level of activity, independent of circadian timing. A fair summary is that there is no agreement on a single locus in the brain where FEOs necessary and sufficient for food-anticipatory rhythms reside. A default position is that many loci are capable of driving FAA ^55– 57^. In his first report on food-anticipatory rhythms, Stephan ^37^ cautioned that “if many oscillators exist which are entrainable by food restriction schedules, it may not be possible to abolish anticipatory activity by selective removal, or interference with, specific organ systems”. That may prove to be true. But the absence of evidence for a defined area analogous to the SCN is not yet sufficiently exhaustive to constitute evidence of absence.

By using the word *organ* rather than *brain*, Stephan intended to include peripheral, non-neural tissues as potential mediators of behavioral rhythms. In the very first report of food anticipation, Richter ^33^ included data on gastric contractions and proposed that “anticipation may be explained by the clock-like functioning of the (stomach)”. In that tradition, Stephan and colleagues targeted the duodenum, autonomic nerves and first-order brainstem nuclei as potential sites of FEOs driving behavior. These efforts were instructive and necessary but ultimately not successful ^43, 48^. The subsequent discovery that bona fide circadian FEOs (cells that express clock gene rhythms that are entrained by daily feeding schedules) are found throughout the body ^10, 17^, combined with a lack of success in defining critical neural loci, has renewed interest in peripheral organs and their outputs as sources of timing information for food-anticipatory behavior.

## Food anticipation and gastric ghrelin

Richter would no doubt have been excited by the discovery of ghrelin, a hormone derived from preproghrelin secreted by gastric oxyntic cells ^58^. Gastric ghrelin secretion is suppressed by food intake and stimulated by fasting and, in its acylated form, binds to the growth hormone secretagogue receptor 1a on cells located in the hypothalamus, substantia nigra, and other brain sites ^59^. It is weakly orexigenic following systemic injection ^60^. In mice, oxyntic cells express circadian cycles of clock gene expression synchronized to mealtime ^61^; ghrelin-secreting cells are therefore FEOs. If these cells, via acyl-ghrelin, provide time signals critical for food anticipation, then ghrelin ligand or receptor KO should eliminate food anticipation. Several groups have conducted this experiment, and the results range from a reduction in the duration of food anticipation ^61^, to a reduced amount of anticipatory activity with no change in duration ^62^, to no significant effect ^63, 64^. The varying results across studies have not been reconciled but could relate to differences in behavioral measures (for example, running wheels and electroencephalogram-based sleep–wake recordings) or other procedural variables. Collectively, the results indicate that ghrelin derived from gastric FEOs is not required for FAA.

A role for ghrelin in initiating food-anticipatory rhythms is also challenged by dissociations between acyl-ghrelin or the gastric clock and FAA during food deprivation and following *ad libitum* food access. Once established, FAA rises and falls with expected mealtime, across multiple days of fasting ^38, 39, 42^. This is a critical, defining attribute of a clock-controlled process. Plasma acyl-ghrelin does not; it rises prior to the next mealtime and then gradually decreases without further cycling ^65, 66^. When food is provided *ad libitum*, rats and mice previously fed in the middle of the light period revert to nocturnal feeding, and daytime FAA rapidly dissipates. Within a few days, the gastric clock has also reset to a nocturnal alignment ^67^. If the animal is then food-deprived, activity reappears at the original scheduled mealtime. This occurs in intact rats in LD or dark–dark (DD) and in SCN-ablated rats, indicating that it is not a “memory” of SCN phase. During food deprivation, gastric clock gene rhythms (expressed by oxyntic ghrelin-secreting cells) remain nocturnally phased ^67^. Finally, rats can anticipate two daily meals; if one is in the light period and the other at night, peripheral clocks assume an intermediate phase or maintain a nocturnal alignment, depending on the relative meal sizes, intermeal interval, and timing within the day ^67– 69^.

These dissociations make clear that ghrelin secreted by gastric FEOs does not directly initiate FAA, but the results do not rule out a role for ghrelin as a participant in phase control of FEOs for behavior which are located elsewhere in the body or brain. This could be explored by measuring the timing of food anticipation on days after a bolus of ghrelin is administered systemically, a number of hours before or after the usual feeding time. If ghrelin can shift FEOs driving FAA, then at one or more phases of these FEOs, an artificial spike of ghrelin should induce a behavioral shift, indicated by a change in the onset of FAA the next day. Injections repeated at the same time each day might induce anticipation, especially if the animal is fasted during those days. Note that experiments that require more than one day of fasting can be carried out only by using adult rats, which, unlike mice, can be food-deprived safely for 3 to 4 days.

## Food anticipation and liver ketone bodies

A large portion of the transcriptome in liver cells exhibits 24 hour rhythmicity ^3^. The timing of these rhythms is controlled by circadian clock genes, which are food-entrainable, and also by direct effects of feeding and fasting cycles independent of clock genes ^17, 22– 25^. Hepatocytes are therefore FEOs, and signals emitted by the liver could propagate food entrainment to other tissues, including the brain. Chavan and colleagues ^70^ reported that global or liver-specific *Per2* KO, but not a neuron-specific *Per2* KO, eliminated daily rhythms of food anticipation in mice fed for 8 hours per day, beginning 4 hours into the daily 12-hour light period. Food anticipation was rescued in liver-specific *Per2* KO mice, but not in global *Per2* KO mice, by virus-mediated constitutive overexpression of *Per2* in the liver. *Per2* KO reduced expression of genes encoding enzymes important for synthesis of ketone bodies by liver cells, and plasma levels of the ketone body β-hydroxybutyrate (βOHB) were reduced during restricted feeding. Ketones provide energy for neurons during fasting, and a daily rhythm of ketone production induced by a feeding schedule could elicit food anticipation if ketone levels regulate neural output ^71^ or clock gene cycling ^72^. Chavan and colleagues ^70^ confirmed that plasma βOHB increases prior to mealtime in day-fed mice and tested a role in food anticipation by using subcutaneous minipumps programmed to release βOHB prior to mealtime in liver-specific and global *Per2* KO mice. This treatment partially rescued FAA. The results were interpreted as evidence that FAA requires *Per2* expression in the liver (although it does not have to be rhythmic) and in one other location (to explain the failure of βOHB to rescue FAA in global *Per2* KO mice) and that βOHB provides timing signals to brain circuits that drive FAA.

Although the combined demonstration of loss and rescue of function makes a compelling narrative, the loss of function requires further study. To induce robust food anticipation, meal durations are conventionally set in the 4- to 6-hour range, or a limited amount of food is provided, to reduce body weight by 10 to 20% ^73^. In the study by Chavan and colleagues, an 8-hour meal duration was used. LeSauter and colleagues ^74^ recently showed that mice overexpressing dopamine 2 receptors (D2Rs) in the striatum fail to anticipate mealtime if food is provided for 8 hours but show robust levels of anticipation, comparable to those of wild-type mice, if the meal duration is 4 or 6 hours. Wild-type mice also showed weak anticipation to the 8-hour duration. Lack of anticipation to the 8-hour meal in D2R-overexpressing mice was attributed to a motivational rather than a clock deficit, which is consistent with other evidence for motivational deficits in these mice. This serves as a cautionary tale that if food anticipation is weak or absent at long meal durations, then the feeding window needs to be narrowed to fully interpret the results.

The dissociations noted between FAA and circulating acyl-ghrelin levels also apply to liver ketones. During extended fasting (in rats), FAA persists with a 24-hour periodicity while plasma ketone levels rise and by the second day remain high ^75^. Therefore, circulating ketones cannot explain the timing of FAA during fasting. Nonetheless, ketones, like ghrelin, could play a role in setting the phase of FEOs in the brain. If so, acute manipulations of ketone levels, at some phases of the food anticipation rhythm, would be expected to shift its timing. Ketone pulses should also be able to shift clock gene cycles in at least some tissues *in vitro*. Chavan and colleagues noted that βOHB did not induce *Per2* expression in the liver, but whether it affects clock gene cycles in other tissues, including neurons, remains to be determined.

An unresolved issue is that, in many studies, contrary to the results reported by Chavan and others ^70 ^, *Per2* KO alone or in combination with KO of *Per1* and *Per3* did not eliminate food anticipation rhythms ^76– 78^. This could be attributable to differences in the mouse lines, or in some methodological details, despite what appear to be similar procedures (meal duration being a notable exception). It would be useful to see mouse lines exchanged across labs and tested using lab-specific procedures.

## Food anticipation and pancreatic insulin

Insulin is secreted by the pancreas in response to glucose ingestion and absorption. Clock gene rhythms in the pancreas entrain to daily meals ^27, 79^. The pancreas thus contains FEOs and emits a hormone that reports mealtime and that has near universal access to cells in the brain and body. A recent study proposed that insulin and insulin-like growth factor-1 (IGF-1) may be universal Zeitgebers for FEOs ^27^. If so, then the pancreas may participate in control of FEOs responsible for food-anticipatory behavioral rhythms ^27^. The study confirmed earlier work that insulin can shift circadian clocks *in vitro* and extended this to an *in vivo* model. *In vitro*, high doses of insulin rapidly increased PER2 levels and shifted the rhythm of PER2::LUC expression in fibroblasts, dissociated cortical neurons, and explants of liver and kidney. In PER2::LUC mice, systemic injections of insulin and glucose induced and shifted the whole organism bioluminescence rhythm. The acute effect of insulin on PER2 levels was mediated by activation of target of rapamycin (TOR), increased phosphoinositide signaling, and microRNA downregulation ^27^.

Two additional findings of note were that, *in vitro*, insulin did not shift the SCN clock but did induce PER2 in fibroblasts lacking *Cry1/Cry2* or *Bmal1*. These observations are consistent with the lack of shifting of SCN clock gene rhythms in food-restricted mice entrained to LD ^10, 29^ and with the failure of *Cry1/Cry2* or *Bmal1* KO to eliminate food anticipation rhythms despite eliminating circadian rhythms when food is available *ad libitum* ^80– 83^. A number of studies have documented the resilience of circadian food anticipation to loss of clock genes in various combinations ^78^. Crosby and colleagues ^27^ propose that feeding cycles, via insulin, can directly drive PER2 cycling in clock gene KO mice. This potentially explains the continued appearance of FAA in clock gene KO mice.

Although the persistence of food-anticipatory rhythms in clock gene KO mice may suggest an entirely novel clock mechanism, changes in circadian properties of FAA in these models do indicate a role for canonical clock genes. For example, FAA in mice lacking *Bmal1* have an expanded (unlimited?) range of entrainment ^82^ (raising the question, if there is no limit, should we call it entrainment or would interval timing be more accurate?). FAA in mice lacking all three *Per* genes appears to have a shortened period of oscillation and entrains best to feeding cycles normally outside of the range of entrainment (for example, 21 hours) ^84^. FAA may be weak or absent in *Rev-erbα* KO mice ^85^. Conceivably, FEOs in mice lacking *Per* genes may cycle because of effects of feeding-related signals on remaining clock genes, including *Bmal1* and *Rev-erbα*.

Crosby and colleagues establish a case for insulin signaling as a potential universal resetting stimulus for FEOs. It is therefore reasonable to infer that this would include FEOs that generate food-anticipatory behavioral rhythms. Genetic ablation of insulin and IGF-1 signaling induces profound metabolic deficits, but pharmacological block is possible using the compound BMS-754807. The authors reasoned that “If insulin and IGF-1 signaling communicates feeding time to cellular circadian clocks throughout the brain and body, then chronic application of BMS-754807 via drinking water should impair entrainment of both molecular and behavioral circadian rhythms to feed-fast cycles *in vivo*” ^27^. To test this, mice entrained to LD with food available *ad libitum* were released into constant light, and food was restricted to the first 8 hours of what was the previous light period, for 8 days, after which food was again provided *ad libitum*. In constant light, LD-entrained activity rhythms free-run with a periodicity usually greater than 24 hours, as shown by the *ad libitum*–fed control group (Figure 7C) ^27^. Food-restricted mice receiving BMS-754807 in drinking water or drinking water alone (vehicle group) also showed a long-period (phase-delaying) activity rhythm. Notably, both vehicle and BMS-754807–treated mice showed substantial activity immediately preceding the mealtime. One interpretation of these data is that insulin does not control the phase of FEOs that drive FAA, but it is also possible that premeal activity was part of the free-running activity rhythm and not the output of FEOs. To examine this further, the chronic BMS-754807 experiment could be repeated on mice (or rats) entrained to LD with food limited to the middle of the light period, when there can be a clear separation between food-anticipatory and nocturnal activity. If BMS-754807–treated mice exhibit a delay in the emergence of FAA or a difference in FAA duration (timing), that would suggest a role for insulin in control of the FEOs that drive behavioral anticipation. It would also be informative to determine whether the rhythm of FAA, once established, can be acutely shifted by a bolus injection of insulin and glucose outside of the usual mealtime.

## Is there a final common clock for circadian anticipation?

Mealtiming exerts a profound influence on circadian clocks throughout the body and brain, some of which control behavior, ensuring that animals can exploit temporal regularities in food availability even when these conflict with a “preferred” chronotype defined when food is available *ad libitum*. The studies highlighted here have identified specific metabolic signals (ghrelin, ketone bodies and insulin) that may participate in the induction of food-anticipatory behavioral rhythms. The available evidence falls short of assigning a necessary or sufficient role for these signals on either the input side or the output side of the food anticipation timing system. It is possible that phase control of this system is multiply determined. Indeed, additional nutrient signaling pathways by which daily cycles of feeding and fasting can regulate clock gene expression have been identified in various cell types ^17– 22^. Synchronization of circadian clocks by food thus appears to involve multiple, tissue-specific or universal input signals acting in parallel or in series to align circadian physiology with mealtimes. Which and how many of these participate in control of behavioral rhythms remain open questions.

The availability of widely broadcast systemic time cues for food entrainment has been taken to suggest that food-anticipatory behavioral rhythms are driven by FEOs distributed across multiple brain circuits that collectively regulate appetitive behavior ^27, 55– 57, 86, 87^. There certainly are many FEOs, in both the brain and the body, but that does not rule out the possibility that there is a “final common” circadian clock that integrates multiple time cues and ultimately sets the phase and period of anticipatory behavioral rhythms. There is ample evidence that animals can anticipate restricted access to other appetitive stimuli, including highly palatable snacks ^88, 89^, water ^90, 91^, salt ^92^, reproductively receptive mates ^93^, and addictive, psychomotor stimulant drugs ^54, 94– 96^. By contrast, scheduled arousal by sleep deprivation, time-restricted running-wheel access, forced running or swimming does not induce anticipatory activity ^97, 98^. Anticipation of water, sex and drugs has been experimentally separated from effects of these schedules on food intake, ruling out spurious food entrainment ^91, 93, 95^. These appetitive stimuli share with food ingestion the ability to strongly activate DA signaling in neural circuits that mediate reward processing, reinforcement learning and habit formation ^99^. Circadian clock genes oscillate in the major components of this system, including the dorsal and ventral striatum, and the timing of these oscillations is shifted by feeding schedules ^86, 100– 102^. In the dorsal striatum, clock gene rhythms are also regulated by DA receptor signaling ^101^. DA-deficient mice do not exhibit appetitive behavior ^103, 104^, but restoring DA signaling selectively in the dorsal striatum is sufficient to rescue these behaviors ^104– 106^, including circadian FAA ^54^. FAA is diminished by DA antagonists ^107, 108^ and by DA receptor 1 KO ^54^ and can be shifted by a DA receptor 2 agonist ^109^. Gastric acyl-ghrelin, pancreatic insulin and hepatic ketone bodies modulate neural activity in midbrain DA neurons and thus converge on DA-receptive circadian oscillators distributed diffusively in striatal reward circuits ^110^. Such oscillators could mediate anticipation of other rewards and, depending on how they are coupled, potentially enable anticipation of more than one daily mealtime or other reward (for example, anticipation of both water and food ^91^ or drug and food ^95^ provided at different times of day).

The concept of a “master”, multioscillatory, DA-responsive circadian clock system for reward anticipation is consistent with the available evidence and makes testable predictions. If DA signaling sets the phase of oscillators that control FAA timing, then it should be possible to shift FAA onset by timed activation of midbrain DA neurons or DA-receptive neurons using chemogenetic or optogenetic tools. It should also be possible to reproduce FAA rhythm phenotypes (for example, loss of limits to entrainment, shortened rhythm period, or failure to emerge) associated with global clock gene KOs (for example, *Bmal1*, *Per1/Per2* and *Rev-Erbα*) by KOs specific to dopaminergic or DA-receptive cell populations in the striatum.

## Concluding thoughts

This article has served as a vehicle to highlight some unresolved issues in mammalian circadian biology, including the formal structure, molecular and cellular substrates and input pathways of the circadian timing system that synchronizes appetitive behavior with daily rhythms in the availability of food or other strong rewards. I conclude with two lines of evidence that link FAA rhythms with ultradian activity rhythms and DA-responsive clocks with psychopathology.

The utility of a circadian system for adjusting rest–activity cycles to times of day most favorable for resource acquisition is self-evident, but it is not unreasonable to speculate that clock entrainment could support addictive behaviors. If episodes of drug, alcohol or junk food consumption are concentrated at a particular time of day (for example, evenings), circadian entrainment processes that serve to reinforce successful daily patterns of reward acquisition could contribute to the emergence, maintenance and relapse of compulsive eating, drinking or drug taking ^111– 113^. Another line of work suggests that ultradian oscillators can induce behavioral rhythms at circadian or longer intervals, depending on DA tone (manipulated, for example, by chronic methamphetamine consumption) and striatal DA levels ^114^. This has been proposed as an animal model of rapid cycling bipolar disorder ^114^. Circadian activity rhythms induced by chronic methamphetamine share several properties with food-anticipatory circadian rhythms, including persistence in rats or mice following SCN ablation or clock gene KOs ^84, 115– 117^. Ultradian rhythms in at least one species, the vole, share with food-anticipatory rhythms in rats the property of being resistant to rhythm period lengthening by chronic consumption of D _2_O in place of H _2_O ^118, 119^. These converging narratives invite speculation that a common pool of DA-regulated oscillators may underlie behavioral rhythmicity in the ultradian and circadian range and behavioral disorders associated with hyperdopaminergic states.

## References

[1] Takahashi JS: Transcriptional architecture of the mammalian circadian clock. *Nat Rev Genet.* 2017;18(3):164–179. 10.1038/nrg.2016.150 27990019 PMC5501165

[2] Hirano A Fu YH Ptáček LJ: The intricate dance of post-translational modifications in the rhythm of life. *Nat Struct Mol Biol.* 2016;23(12):1053–1060. 10.1038/nsmb.3326 27922612

[3] Kornmann B Schaad O Bujard H: System-Driven and Oscillator-Dependent Circadian Transcription in Mice with a Conditionally Active Liver Clock. *PLoS Biol.* 2007;5(2):e34. 10.1371/journal.pbio.0050034 17298173 PMC1783671

[4] Yeung J Naef F: Rhythms of the Genome: Circadian Dynamics from Chromatin Topology, Tissue-Specific Gene Expression, to Behavior. *Trends Genet.* 2018;34(12):915–926. 10.1016/j.tig.2018.09.005 30309754

[5] Golombek DA Rosenstein RE: Physiology of Circadian Entrainment. *Physiol Rev.* 2010;90(3):1063–102. 10.1152/physrev.00009.2009 20664079

[6] Rosbash M: A 50-Year Personal Journey: Location, Gene Expression, and Circadian Rhythms. *Cold Spring Harb Perspect Biol.* 2017;9(12):pii: a032516. 10.1101/cshperspect.a032516 28600396 PMC5710103

[7] Young MW: Time Travels: A 40-Year Journey from Drosophila's Clock Mutants to Human Circadian Disorders (Nobel Lecture). *Angew Chem Int Ed Engl.* 2018;57(36):11532–11539. 10.1002/anie.201803337 30003624

[8] Patton AP Hastings MH: The suprachiasmatic nucleus. *Curr Biol.* 2018;28(15):R816–R822. 10.1016/j.cub.2018.06.052 30086310

[9] Abe M Herzog ED Yamazaki S: Circadian Rhythms in Isolated Brain Regions. *J Neurosci.* 2002;22(1):350–6. 10.1523/JNEUROSCI.22-01-00350.2002 11756518 PMC6757616

[10] Schibler U: The 2008 Pittendrigh/Aschoff Lecture: Peripheral Phase Coordination in the Mammalian Circadian Timing System. *J Biol Rhythms.* 2009;24(1):3–15. 10.1177/0748730408329383 19150925

[11] Guilding C Piggins HD: Challenging the omnipotence of the suprachiasmatic timekeeper: Are circadian oscillators present throughout the mammalian brain? *Eur J Neurosci.* 2007;25(11):3195–216. 10.1111/j.1460-9568.2007.05581.x 17552989

[12] Guilding C Hughes AT Brown TM: A riot of rhythms: Neuronal and glial circadian oscillators in the mediobasal hypothalamus. *Mol Brain.* 2009;2:28. 10.1186/1756-6606-2-28 19712475 PMC2745382

[13] Guilding C Hughes AT Piggins HD: Circadian oscillators in the epithalamus. *Neuroscience.* 2010;169(4):1630–9. 10.1016/j.neuroscience.2010.06.015 20547209 PMC2928449

[14] Brown AJ Pendergast JS Yamazaki S: Peripheral Circadian Oscillators. *Yale J Biol Med.* 2019;92(2):327–335. 31249493 PMC6585520

[15] Morin LP: Neuroanatomy of the extended circadian rhythm system. *Exp Neurol.* 2013;243:4–20. 10.1016/j.expneurol.2012.06.026 22766204 PMC3498572

[16] Kalsbeek A Palm IF La Fleur SE: SCN outputs and the hypothalamic balance of life. *J Biol Rhythms.* 2006;21(6):458–69. 10.1177/0748730406293854 17107936

[17] Schibler U Gotic I Saini C: Clock-Talk: Interactions between Central and Peripheral Circadian Oscillators in Mammals. *Cold Spring Harb Symp Quant Biol.* 2016;80:223–32. 10.1101/sqb.2015.80.027490 26683231

[18] Hirota T Okano T Kokame K: Glucose down-regulates *Per1* and *Per2* mRNA levels and induces circadian gene expression in cultured Rat-1 fibroblasts. *J Biol Chem.* 2002;277(46):44244–51. 10.1074/jbc.M206233200 12213820

[19] Lamia KA Sachdeva UM DiTacchio L: AMPK Regulates the Circadian Clock by Cryptochrome Phosphorylation and Degradation. *Science.* 2009;326(5951):437–40. 10.1126/science.1172156 19833968 PMC2819106

[20] Nakahata Y Kaluzova M Grimaldi B: The NAD+-Dependent Deacetylase SIRT1 Modulates CLOCK-Mediated Chromatin Remodeling and Circadian Control. *Cell.* 2008;134(2):329–40. 10.1016/j.cell.2008.07.002 18662547 PMC3526943

[21] Asher G Gatfield D Stratmann M: SIRT1 Regulates Circadian Clock Gene Expression through PER2 Deacetylation. *Cell.* 2008;134(2):317–28. 10.1016/j.cell.2008.06.050 18662546

[22] Asher G Reinke H Altmeyer M: Poly(ADP-Ribose) Polymerase 1 Participates in the Phase Entrainment of Circadian Clocks to Feeding. *Cell.* 2010;142(6):943–53. 10.1016/j.cell.2010.08.016 20832105

[23] Balsalobre A Brown SA Marcacci L: Resetting of Circadian Time in Peripheral Tissues by Glucocorticoid Signaling. *Science.* 2000;289(5488):2344–7. 10.1126/science.289.5488.2344 11009419

[24] Tahara Y Otsuka M Fuse Y: Refeeding after fasting elicits insulin-dependent regulation of *Per2* and *Rev-erbα* with shifts in the liver clock. *J Biol Rhythms.* 2011;26(3):230–40. 10.1177/0748730411405958 21628550

[25] Yamajuku D Inagaki T Haruma T: Real-time monitoring in three-dimensional hepatocytes reveals that insulin acts as a synchronizer for liver clock. *Sci Rep.* 2012;2:439. 10.1038/srep00439 22666542 PMC3365280

[26] Chaves I van der Horst GTJ Schellevis R: Insulin-FOXO3 signaling modulates circadian rhythms via regulation of *clock* transcription. *Curr Biol.* 2014;24(11):1248–55. 10.1016/j.cub.2014.04.018 24856209

[27] Crosby P Hamnett R Putker M: Insulin/IGF-1 Drives PERIOD Synthesis to Entrain Circadian Rhythms with Feeding Time. *Cell.* 2019;177(4):896–909.e20. 10.1016/j.cell.2019.02.017 31030999 PMC6506277

[28] Landgraf D Tsang AH Leliavski A: Oxyntomodulin regulates resetting of the liver circadian clock by food. *eLife.* 2015;4:e06253. 10.7554/eLife.06253 25821984 PMC4426666

[29] Damiola F Le Minh N Preitner N: Restricted feeding uncouples circadian oscillators in peripheral tissues from the central pacemaker in the suprachiasmatic nucleus. *Genes Dev.* 2000;14(23):2950–61. 10.1101/gad.183500 11114885 PMC317100

[30] Hara R Wan K Wakamatsu H: Restricted feeding entrains liver clock without participation of the suprachiasmatic nucleus. *Genes Cells.* 2001;6(3):269–78. 10.1046/j.1365-2443.2001.00419.x 11260270

[31] Stokkan KA Yamazaki S Tei H: Entrainment of the circadian clock in the liver by feeding. *Science.* 2001;291(5503):490–3. 10.1126/science.291.5503.490 11161204

[32] Coomans CP Ramkisoensing A Meijer JH: The suprachiasmatic nuclei as a seasonal clock. *Front Neuroendocrinol.* 2015;37:29–42. 10.1016/j.yfrne.2014.11.002 25451984

[33] Richter CP: A behavioristic study of the activity of the rat. Baltimore: Williams & Wilkins Company;1922 10.5962/bhl.title.151527

[34] Bolles RC Moot SA: The rat's anticipation of two meals a day. *J Comp Physiol Psychol.* 1973;83(3):510–4. 10.1037/h0034666 4715311

[35] Bolles RC de Lorge J: The rat's adjustment to a-diurnal feeding cycles. *J Comp Physiol Psychol.* 1962;55:760–2. 10.1037/h0046716 13968622

[36] Bolles RC Stokes LW: Rat's anticipation of diurnal and a-diurnal feeding. *J Comp Physiol Psychol.* 1965;60(2):290–4. 10.1037/h0022308 5832364

[37] Stephan FK Swann JM Sisk CL: Entrainment of circadian rhythms by feeding schedules in rats with suprachiasmatic lesions. *Behav Neural Biol.* 1979;25(4):545–54. 10.1016/s0163-1047(79)90332-7 464989

[38] Stephan FK Swann JM Sisk CL: Anticipation of 24-hr feeding schedules in rats with lesions of the suprachiasmatic nucleus. *Behav Neural Biol.* 1979;25(3):346–63. 10.1016/s0163-1047(79)90415-1 464979

[39] Boulos Z Rosenwasser AM Terman M: Feeding schedules and the circadian organization of behavior in the rat. *Behav Brain Res.* 1980;1(1):39–65. 10.1016/0166-4328(80)90045-5 7284080

[40] Boulos Z Terman M: Food availability and daily biological rhythms. *Neurosci Biobehav Rev.* 1980;4(2):119–31. 10.1016/0149-7634(80)90010-x 6106914

[41] Aschoff J: Activity in anticipation and in succession of a daily meal. *Boll Soc Ital Biol Sper.* 1991;67(3):213–28. 1930896

[42] Mistlberger RE: Circadian food-anticipatory activity: formal models and physiological mechanisms. *Neurosci Biobehav Rev.* 1994;18(2):171–95. 10.1016/0149-7634(94)90023-x 8058212

[43] Stephan FK: The "other" circadian system: food as a Zeitgeber. *J Biol Rhythms.* 2002;17(4):284–92. 12164245 10.1177/074873040201700402

[44] Stephan FK: Circadian rhythm dissociation induced by periodic feeding in rats with suprachiasmatic lesions. *Behav Brain Res.* 1983;7(1):81–98. 10.1016/0166-4328(83)90006-2 6824529

[45] Stephan FK: Forced dissociation of activity entrained to T cycles of food access in rats with suprachiasmatic lesions. *J Biol Rhythms.* 1989;4(4):467–79. 10.1177/074873048900400406 2519607

[46] Pittendrigh CS Daan S: A functional analysis of circadian pacemakers in nocturnal rodents. *J Comp Physiol.* 1976;106(3):333–355. 10.1007/BF01417860

[47] Meijer JH Daan S Overkamp GJ: The two-oscillator circadian system of tree shrews ( *Tupaia belangeri*) and its response to light and dark pulses. *J Biol Rhythms.* 1990;5(1):1–16. 10.1177/074873049000500101 2133115

[48] Davidson AJ: Lesion studies targeting food-anticipatory activity. *Eur J Neurosci.* 2009;30(9):1658–64. 10.1111/j.1460-9568.2009.06961.x 19863659

[49] Mistlberger RE: Neurobiology of food anticipatory circadian rhythms. *Physiol Behav.* 2011;104(4):535–45. 10.1016/j.physbeh.2011.04.015 21527266

[50] Challet E Mendoza J Dardente H: Neurogenetics of food anticipation. *Eur J Neurosci.* 2009;30(9):1676–87. 10.1111/j.1460-9568.2009.06962.x 19863658

[51] Butler AA Girardet C Mavrikaki M: A Life without Hunger: The Ups (and Downs) to Modulating Melanocortin-3 Receptor Signaling. *Front Neurosci.* 2017;11:128. 10.3389/fnins.2017.00128 28360832 PMC5352694

[52] Davidson AJ Cappendijk SL Stephan FK: Feeding-entrained circadian rhythms are attenuated by lesions of the parabrachial region in rats. *Am J Physiol Regul Integr Comp Physiol.* 2000;278(5):R1296–304. 10.1152/ajpregu.2000.278.5.R1296 10801300

[53] Mendoza J Pevet P Felder-Schmittbuhl MP: The cerebellum harbors a circadian oscillator involved in food anticipation. *J Neurosci.* 2010;30(5):1894–904. 10.1523/JNEUROSCI.5855-09.2010 20130198 PMC6634001

[54] Gallardo CM Darvas M Oviatt M: Dopamine receptor 1 neurons in the dorsal striatum regulate food anticipatory circadian activity rhythms in mice. *eLife.* 2014;3:e03781. 10.7554/eLife.03781 25217530 PMC4196120

[55] Escobar C Cailotto C Angeles-Castellanos M: Peripheral oscillators: the driving force for food-anticipatory activity. *Eur J Neurosci.* 2009;30(9):1665–75. 10.1111/j.1460-9568.2009.06972.x 19878276

[56] Carneiro BT Araujo JF: The food-entrainable oscillator: a network of interconnected brain structures entrained by humoral signals? *Chronobiol Int.* 2009;26(7):1273–89. 10.3109/07420520903404480 19916831

[57] Challet E Mendoza J: Metabolic and reward feeding synchronises the rhythmic brain. *Cell Tissue Res.* 2010;341(1):1–11. 10.1007/s00441-010-1001-9 20563601

[58] Guan XM Yu H Palyha OC: Distribution of mRNA encoding the growth hormone secretagogue receptor in brain and peripheral tissues. *Brain Res Mol Brain Res.* 1997;48(1):23–9. 10.1016/s0169-328x(97)00071-5 9379845

[59] Kojima M Hosoda H Date Y: Ghrelin is a growth-hormone-releasing acylated peptide from stomach. *Nature.* 1999;402(6762):656–60. 10.1038/45230 10604470

[60] Nakazato M Murakami N Date Y: A role for ghrelin in the central regulation of feeding. *Nature.* 2001;409(6817):194–8. 10.1038/35051587 11196643

[61] LeSauter J Hoque N Weintraub M: Stomach ghrelin-secreting cells as food-entrainable circadian clocks. *Proc Natl Acad Sci U S A.* 2009;106(32):13582–7. 10.1073/pnas.0906426106 19633195 PMC2726387

[62] Blum ID Patterson Z Khazall R: Reduced anticipatory locomotor responses to scheduled meals in ghrelin receptor deficient mice. *Neuroscience.* 2009;164(2):351–9. 10.1016/j.neuroscience.2009.08.009 19666088 PMC2996828

[63] Gunapala KM Gallardo CM Hsu CT: Single gene deletions of orexin, leptin, neuropeptide Y, and ghrelin do not appreciably alter food anticipatory activity in mice. *PLoS One.* 2011;6(3):e18377. 10.1371/journal.pone.0018377 21464907 PMC3065493

[64] Szentirmai E Kapás L Sun Y: Restricted feeding-induced sleep, activity, and body temperature changes in normal and preproghrelin-deficient mice. *Am J Physiol Regul Integr Comp Physiol.* 2010;298(2):R467–77. 10.1152/ajpregu.00557.2009 19939974 PMC2828180

[65] Liu J Prudom CE Nass R: Novel ghrelin assays provide evidence for independent regulation of ghrelin acylation and secretion in healthy young men. *J Clin Endocrinol Metab.* 2008;93(5):1980–7. 10.1210/jc.2007-2235 18349056 PMC2386282

[66] Kirchner H Gutierrez JA Solenberg PJ: GOAT links dietary lipids with the endocrine control of energy balance. *Nat Med.* 2009;15(7):741–5. 10.1038/nm.1997 19503064 PMC2789701

[67] Davidson AJ Poole AS Yamazaki S: Is the food-entrainable circadian oscillator in the digestive system? *Genes Brain Behav.* 2003;2(1):32–9. 10.1034/j.1601-183x.2003.00005.x 12882317

[68] Hirao A Nagahama H Tsuboi T: Combination of starvation interval and food volume determines the phase of liver circadian rhythm in Per2::Luc knock-in mice under two meals per day feeding. *Am J Physiol Gastrointest Liver Physiol.* 2010;299(5):G1045–53. 10.1152/ajpgi.00330.2010 20847299

[69] Patton DF Katsuyama AM Pavlovski I: Circadian mechanisms of food anticipatory rhythms in rats fed once or twice daily: clock gene and endocrine correlates. *PLoS One.* 2014;9(12):e112451. 10.1371/journal.pone.0112451 25502949 PMC4263600

[70] Chavan R Feillet C Costa SS: Liver-derived ketone bodies are necessary for food anticipation. *Nat Commun.* 2016;7:10580. 10.1038/ncomms10580 26838474 PMC4742855

[71] Carneiro L Geller S Fioramonti X: Evidence for hypothalamic ketone body sensing: impact on food intake and peripheral metabolic responses in mice. *Am J Physiol Endocrinol Metab.* 2016;310(2):E103–15. 10.1152/ajpendo.00282.2015 26530151

[72] Genzer Y Dadon M Burg C: Ketogenic diet delays the phase of circadian rhythms and does not affect AMP-activated protein kinase (AMPK) in mouse liver. *Mol Cell Endocrinol.* 2015;417:124–30. 10.1016/j.mce.2015.09.012 26408964

[73] Mistlberger RE: Food-anticipatory circadian rhythms: concepts and methods. *Eur J Neurosci.* 2009;30(9):1718–29. 10.1111/j.1460-9568.2009.06965.x 19878279

[74] LeSauter J Balsam PD Simpson EH: Overexpression of striatal D2 receptors reduces motivation thereby decreasing food anticipatory activity. *Eur J Neurosci.* 2018. 10.1111/ejn.14219 30362616 PMC7783547

[75] Newman JC Verdin E: β-Hydroxybutyrate: A Signaling Metabolite. *Annu Rev Nutr.* 2017;37:51–76. 10.1146/annurev-nutr-071816-064916 28826372 PMC6640868

[76] Storch KF Weitz CJ: Daily rhythms of food-anticipatory behavioral activity do not require the known circadian clock. *Proc Natl Acad Sci U S A.* 2009;106(16):6808–13. 10.1073/pnas.0902063106 19366674 PMC2666092

[77] Pendergast JS Wendroth RH Stenner RC: *mPeriod2* *^Brdm1^* and other single *Period* mutant mice have normal food anticipatory activity. *Sci Rep.* 2017;7(1):15510. 10.1038/s41598-017-15332-6 29138421 PMC5686205

[78] Pendergast JS Yamazaki S: The Mysterious Food-Entrainable Oscillator: Insights from Mutant and Engineered Mouse Models. *J Biol Rhythms.* 2018;33(5):458–474. 10.1177/0748730418789043 30033846 PMC6693510

[79] Petrenko V Philippe J Dibner C: Time zones of pancreatic islet metabolism. *Diabetes Obes Metab.* 2018;20 Suppl 2:116–126. 10.1111/dom.13383 30230177

[80] Iijima M Yamaguchi S van der Horst GTJ: Altered food-anticipatory activity rhythm in *Cryptochrome*-deficient mice. *Neurosci Res.* 2005;52(2):166–73. 10.1016/j.neures.2005.03.003 15893577

[81] Mendoza J Albrecht U Challet E: Behavioural food anticipation in clock genes deficient mice: confirming old phenotypes, describing new phenotypes. *Genes Brain Behav.* 2010;9(5):467–77. 10.1111/j.1601-183X.2010.00576.x 20180860

[82] Takasu NN Kurosawa G Tokuda IT: Circadian regulation of food-anticipatory activity in molecular clock-deficient mice. *PLoS One.* 2012;7(11):e48892. 10.1371/journal.pone.0048892 23145013 PMC3492221

[83] Pendergast JS Nakamura W Friday RC: Robust food anticipatory activity in BMAL1-deficient mice. *PLoS One.* 2009;4(3):e4860. 10.1371/journal.pone.0004860 19300505 PMC2654093

[84] Pendergast JS Oda GA Niswender KD: Period determination in the food-entrainable and methamphetamine-sensitive circadian oscillator(s). *Proc Natl Acad Sci U S A.* 2012;109(35):14218–23. 10.1073/pnas.1206213109 22891330 PMC3435193

[85] Delezie J Dumont S Sandu C: *Rev-erbα* in the brain is essential for circadian food entrainment. *Sci Rep.* 2016;6:29386. 10.1038/srep29386 27380954 PMC4933951

[86] Verwey M Amir S: Food-entrainable circadian oscillators in the brain. *Eur J Neurosci.* 2009;30(9):1650–7. 10.1111/j.1460-9568.2009.06960.x 19863660

[87] Feillet CA Albrecht U Challet E: "Feeding time" for the brain: a matter of clocks. *J Physiol Paris.* 2006;100(5–6):252–60. 10.1016/j.jphysparis.2007.05.002 17629684

[88] Angeles-Castellanos M Salgado-Delgado R Rodríguez K: Expectancy for food or expectancy for chocolate reveals timing systems for metabolism and reward. *Neuroscience.* 2008;155(1):297–307. 10.1016/j.neuroscience.2008.06.001 18585440

[89] Hsu CT Patton DF Mistlberger RE: Palatable meal anticipation in mice. *PLoS One.* 2010;5(9): pii: e12903. 10.1371/journal.pone.0012903 20941366 PMC2948008

[90] Bolles RC: Anticipatory general activity in thirsty rats. *J Comp Physiol Psychol.* 1968;65(3):511–3. 10.1037/h0025822 5667394

[91] Mistlberger RE: Anticipatory Activity Rhythms under Daily Schedules of Water Access in the Rat. *J Biol Rhythms.* 1992;7(2):149–60. 10.1177/074873049200700206 1611130

[92] Rosenwasser AM Schulkin J Adler NT: Circadian wheel-running activity of rats under schedules of limited daily access to salt. *Chronobiol Int.* 1985;2(2):115–9. 10.3109/07420528509055550 3870841

[93] Landry GJ Opiol H Marchant EG: Scheduled Daily Mating Induces Circadian Anticipatory Activity Rhythms in the Male Rat. *PLoS One.* 2012;7(7):e40895. 10.1371/journal.pone.0040895 22848408 PMC3405034

[94] Mohawk JA Pezuk P Menaker M: Methamphetamine and dopamine receptor D1 regulate entrainment of murine circadian oscillators. *PLoS One.* 2013;8(4):e62463. 10.1371/journal.pone.0062463 23626822 PMC3633847

[95] Jansen HT Sergeeva A Stark G: Circadian Discrimination of Reward: Evidence for Simultaneous Yet Separable Food- and Drug-Entrained Rhythms in the Rat. *Chronobiol Int.* 2012;29(4):454–68. 10.3109/07420528.2012.667467 22475541

[96] Juárez-Portilla C Pitter M Kim RD: Brain Activity during Methamphetamine Anticipation in a Non-Invasive Self-Administration Paradigm in Mice. *eNeuro.* 2018;5(2): pii: ENEURO.0433-17.2018. 10.1523/ENEURO.0433-17.2018 29632871 PMC5889482

[97] Bolles RC Riley AL Cantor MB: The rat's failure to anticipate regularly scheduled daily shock. *Behav Biol.* 1974;11(3):365–72. 10.1016/s0091-6773(74)90633-6 4416448

[98] Webb IC Antle MC Mistlberger RE: Regulation of circadian rhythms in mammals by behavioral arousal. *Behav Neurosci.* 2014;128(3):304–25. 10.1037/a0035885 24773430

[99] Volkow ND Wise RA Baler R: The dopamine motive system: Implications for drug and food addiction. *Nat Rev Neurosci.* 2017;18(12):741–752. 10.1038/nrn.2017.130 29142296

[100] Wakamatsu H Yoshinobu Y Aida R: Restricted-feeding-induced anticipatory activity rhythm is associated with a phase-shift of the expression of *mPer1* and *mPer2* mRNA in the cerebral cortex and hippocampus but not in the suprachiasmatic nucleus of mice. *Eur J Neurosci.* 2001;13(6):1190–6. 10.1046/j.0953-816x.2001.01483.x 11285016

[101] Hood S Cassidy P Cossette MP: Endogenous Dopamine Regulates the Rhythm of Expression of the Clock Protein PER2 in the Rat Dorsal Striatum via Daily Activation of D2 Dopamine Receptors. *J Neurosci.* 2010;30(42):14046–58. 10.1523/JNEUROSCI.2128-10.2010 20962226 PMC6634752

[102] Verwey M Dhir S Amir S: Circadian influences on dopamine circuits of the brain: regulation of striatal rhythms of clock gene expression and implications for psychopathology and disease [version 1; peer review: 2 approved]. *F1000Res.* 2016;5: pii: F1000 Faculty Rev-2062. 10.12688/f1000research.9180.1 27635233 PMC5007753

[103] Zhou Q-Y, Palmiter RD: Dopamine-deficient mice are severely hypoactive, adipsic, and aphagic. *Cell.* 1995;83(7):1197–209. 10.1016/0092-8674(95)90145-0 8548806

[104] Szczypka MS Kwok K Brot MD: Dopamine Production in the Caudate Putamen Restores Feeding in Dopamine-Deficient Mice. *Neuron.* 2001;30(3):819–28. 10.1016/s0896-6273(01)00319-1 11430814

[105] Robinson S Sotak BN During MJ: Local dopamine production in the dorsal striatum restores goal-directed behavior in dopamine-deficient mice. *Behav Neurosci.* 2006;120(1):196–200. 10.1037/0735-7044.120.1.000 16492130

[106] Palmiter RD: Dopamine Signaling in the Dorsal Striatum Is Essential for Motivated Behaviors: lessons from dopamine-deficient mice. *Ann N Y Acad Sci.* 2008;1129:35–46. 10.1196/annals.1417.003 18591467 PMC2720267

[107] Liu YY Liu TY Qu WM: Dopamine is involved in food-anticipatory activity in mice. *J Biol Rhythms.* 2012;27(5):398–409. 10.1177/0748730412455913 23010662

[108] Mistlberger RE Mumby DG: The limbic system and food-anticipatory circadian rhythms in the rat: ablation and dopamine blocking studies. *Behav Brain Res.* 1992;47(2):159–68. 10.1016/s0166-4328(05)80122-6 1590946

[109] Smit AN Patton DF Michalik M: Dopaminergic regulation of circadian food anticipatory activity rhythms in the rat. *PLoS One.* 2013;8(11):e82381. 10.1371/journal.pone.0082381 24312417 PMC3843722

[110] de Lartigue G McDougle M: Dorsal striatum dopamine oscillations: Setting the pace of food anticipatory activity. *Acta Physiol (Oxf).* 2019;225(1):e13152. 10.1111/apha.13152 29920950 PMC6301124

[111] Davidson AJ Tataroglu Ö Menaker M: Circadian Effects of Timed Meals (and Other Rewards). *Methods Enzymol.*In: *Circadian Rhythms.*Elsevier;2005;393:509–523. 10.1016/S0076-6879(05)93026-7 15817309

[112] Webb IC: Circadian Rhythms and Substance Abuse: Chronobiological Considerations for the Treatment of Addiction. *Curr Psychiatry Rep.* 2017;19(2):12. 10.1007/s11920-017-0764-z 28188587

[113] DePoy LM McClung CA Logan RW: Neural Mechanisms of Circadian Regulation of Natural and Drug Reward. *Neural Plast.* 2017;2017:5720842. 10.1155/2017/5720842 29359051 PMC5735684

[114] Blum ID Zhu L Moquin L: A highly tunable dopaminergic oscillator generates ultradian rhythms of behavioral arousal. *eLife.* 2014;3:e05105. 10.7554/eLife.05105 25546305 PMC4337656

[115] Honma K Honma S: The SCN-independent clocks, methamphetamine and food restriction. *Eur J Neurosci.* 2009;30(9):1707–17. 10.1111/j.1460-9568.2009.06976.x 19878275

[116] Mohawk JA Baer ML Menaker M: The methamphetamine-sensitive circadian oscillator does not employ canonical clock genes. *Proc Natl Acad Sci U S A.* 2009;106(9):3519–24. 10.1073/pnas.0813366106 19204282 PMC2651344

[117] Salaberry NL Mateo M Mendoza J: The Clock Gene *Rev-Erbα* Regulates Methamphetamine Actions on Circadian Timekeeping in the Mouse Brain. *Mol Neurobiol.* 2017;54(7):5327–5334. 10.1007/s12035-016-0076-z 27581301

[118] Mistlberger RE Marchant EG Kippin TE: Food-entrained circadian rhythms in rats are insensitive to deuterium oxide. *Brain Res.* 2001;919(2):283–91. 10.1016/s0006-8993(01)03042-6 11701140

[119] Gerkema MP Daan S Wilbrink M: Phase control of ultradian feeding rhythms in the common vole (Microtus arvalis): The roles of light and the circadian system. *J Biol Rhythms.* 1993;8(2):151–71. 10.1177/074873049300800205 8369551

